# Effects of Task-Oriented Training on Gait Outcomes and Balance in Individuals with Stroke: A Systematic Review and Meta-Analysis of Randomized Controlled Trials

**DOI:** 10.3390/jcm14248766

**Published:** 2025-12-11

**Authors:** Myoung-Ho Lee, Do-Youn Lee

**Affiliations:** 1 Department of Rehabilitation Sciences, Graduate School, Daegu University, Jillyang, Gyeongsan 38453, Gyeongbuk, Republic of Korea; hotayaaa@gmail.com; 2 College of General Education, Kookmin University, Seoul 02707, Seongbuk, Republic of Korea; 3 Korea Vestibular Rehabilitation Therapy Association, Jilyang, Gyeongsan 38453, Gyeongbuk, Republic of Korea

**Keywords:** balance, gait, stroke, task-oriented training

## Abstract

**Background/Objectives:** Task-oriented training (TOT) is a functional, goal-directed rehabilitation approach that promotes motor recovery after stroke through repetitive, task-specific practice; however, its overall effects on gait and balance in stroke survivors remain unclear. This systematic review- and meta-analysis-based study aims to evaluate the effects of TOT on gait and balance in patients with stroke. **Methods**: Comprehensive searches of PubMed, Embase, Web of Science, the Cochrane Library, and Scopus were performed. Eligible studies involving TOT interventions in patients with stroke were included, and methodological quality was assessed using the PEDro scale. A random-effects meta-analysis was then performed to estimate pooled effect sizes. **Results**: In total, 17 randomized controlled trials involving 888 participants were included. Compared with control interventions, TOT significantly improved gait speed (standardized mean difference [SMD] = 0.48, 95% confidence interval [CI]: 0.27–0.69, *p* < 0.0001), gait endurance (SMD = 0.49, 95% CI: 0.27–0.71, *p* < 0.0001), Berg Balance Scale (BBS) (SMD = 0.45, 95% CI: 0.08–0.82, *p* = 0.02), and timed up and go test performance (SMD = −0.28, 95% CI: −0.47 to −0.09, *p* = 0.003). Subgroup analysis of the BBS revealed differences based on stroke phase. **Conclusions**: Task-oriented training effectively improves gait and balance in stroke survivors and should be considered a key component of post-stroke rehabilitation. Future studies should explore its long-term effects and determine optimal training parameters according to stroke phase and patient characteristics.

## 1. Introduction

Stroke remains a leading cause of death and disability worldwide, with its incidence continuing to rise as populations age [1]. It is classified as either ischemic or hemorrhagic, with symptoms and prognosis varying depending on lesion type and location [2]. Most stroke survivors experience motor and cognitive impairments that limit walking, balance, and daily activities [3].

Gait and balance deficits increase the risk of falls, reduce independence, and diminish quality of life [4]. Since gait speed and distance are core rehabilitation goals, and balance is essential for gait stability, both are considered critical outcomes in post-stroke recovery [5].

Conventional gait training focuses on repetitive walking exercise, muscle strengthening, and orthotic use, whereas balance training emphasizes movement strategies to maintain posture and control the center of gravity of the body [6]. However, these approaches have limitations because they lack task specificity and adaptability to real-world environments [7]. Studies also report that conventional gait training alone is insufficient for improving overall balance [8].

In contrast, task-oriented training (TOT) promotes sensorimotor integration and adaptation to environmental variability through repetitive, goal-directed functional tasks. It enhances patient engagement, thereby strengthening motor learning and functional recovery [9,10].

Growing evidence suggests that task-oriented activities such as gait and balance training provide greater benefits for functional recovery than simple repetitive exercises. In addition to conventional task-oriented approaches, recent studies have highlighted the role of robotic-assisted task-oriented rehabilitation in stroke recovery. Robotic systems can provide high-intensity, repetitive, and task-specific training while reducing physical burden on therapists and increasing patient engagement. These robotic-assisted programs have demonstrated potential benefits for improving gait and balance outcomes [11,12]. A systematic review comparing intervention methods in patients with stroke reports that virtual reality-based gait training significantly improves specific balance outcomes [13]. Virtual reality (VR)–based rehabilitation provides enriched multisensory input, real-time feedback, and task variability that facilitate motor learning and neural plasticity, thereby enhancing functional mobility and balance recovery in stroke survivors [14]. Similarly, a meta-analysis of exercise interventions in patients with chronic stroke reports positive effects on mobility, balance, and gait [15]. However, exercise-based interventions also have limitations. For example, treadmill-based gait training improves walking distance, but some studies report no significant improvement in gait speed or balance [16].

A meta-analysis of rehabilitation strategies for improving balance reports that core stability training, balance exercises, and combined interventions are effective [17]. However, many studies vary considerably in intervention methods and task specificity, complicating effect comparisons. Furthermore, some studies focus on a single outcome (either gait or balance), limiting the assessment of overall effectiveness.

Therefore, this study aims to quantitatively evaluate the effects of task-directed training on gait outcomes and balance in patients with stroke, focusing on randomized controlled trials (RCTs). By exploring how intervention characteristics (e.g., session frequency, intervention duration) and disease stage (acute/subacute/chronic) influence effect size, this study seeks to provide clearer evidence to guide rehabilitation practice.

## 2. Materials and Methods

This systematic review followed the Preferred Reporting Items for Systematic Reviews and Meta-Analysis (PRISMA) 2020 guidelines, and the completed PRISMA checklist is provided in the Supplementary Material [18,19].

### 2.1. Search Strategy and Data Resource

A comprehensive literature search was conducted between May and June 2025 across EMBASE, Ovid-LWW, Scopus, PubMed, and CINAHL databases, including only articles published in English.

The primary search terms were “stroke” and “task-oriented training,” combined with the Boolean operators “AND” and “OR.”

### 2.2. Eligibility and Exclusion Criteria

Eligibility criteria were defined using the patient, intervention, comparison, outcome (PICO) framework as follows:

- Patients (P): Patients with a stroke (except where a person without disability acts as a person with disabilities);
- Intervention (I): TOT;
- Comparison (C): Conventional therapy or other rehabilitation interventions;
- Outcome (O): Gait and balance outcomes.

Studies were excluded if they were reviews, meta-analyses, letters, or conference proceedings, or if they did not involve stroke survivors. Articles lacking details on interventions, results, or full-text availability were also excluded.

### 2.3. Screening, Selection, and Extraction Process

This review followed the PRISMA 2020 guidelines for study selection. Two researchers independently screened and extracted data using electronic databases, applying predefined inclusion and exclusion criteria. Duplicate and irrelevant records were removed during the identification phase. Titles and abstracts were first reviewed to identify eligible studies, followed by full-text assessment to confirm final inclusion based on the study objectives.

### 2.4. Data Extraction

The data extraction form was developed following the Cochrane Handbook for Systematic Reviews of Interventions [20]. Two independent reviewers (M.-H.L. and D.-Y.L.) pilot-tested the form on five randomly selected studies to verify extraction accuracy [21]. All extracted data were recorded and managed using Microsoft Excel 365. The data extracted included authors, publication year, study design, stroke type, sample size, sex, mean age, stroke stage, intervention method, control group, gait- and balance-related outcomes, measurement type, unit, intention-to-treat (ITT) analysis, missing data handling, and dropout rate.

### 2.5. Risk of Bias Assessment

The methodological quality of the selected studies was assessed using the Physiotherapy Evidence Database (PEDro) scale [22]. The PEDro scale is an established tool for evaluating the quality of clinical interventions and comprises 11 criteria: clearly defined eligibility, random allocation, concealed allocation, baseline group comparability, blinding of participants, therapists, and assessors, dropout rate below 15%, ITT analysis, between-group statistical comparisons, and reporting of point estimates and variability.

### 2.6. Reporting Bias Assessment

A meta-analysis was conducted when ten or more studies examined the same outcome, and funnel plots were used to assess small-study effects and publication bias [23]. Publication bias was further evaluated using the Egger regression test in R-Studio, with a *p*-value < 0.05 indicating statistical significance [24].

### 2.7. Statistical Analysis (Synthesis Methods)

A meta-analysis was conducted using Review Manager v5.4 to synthesize the results.

Heterogeneity and significance were visually interpreted using forest plots. The inverse variance method was used to calculate the standardized mean difference (SMD) for continuous outcomes [25]. The overall effect of TOT on lower limb function was evaluated using the Z-statistic at a significance level of *p* < 0.05. Effect sizes were expressed as the SMD or Cohen’s d, with Hedge’s g also calculated as an adjusted estimate [26]. Since Cohen’s d can overestimate effect sizes in small samples [27], Hedge’s g was calculated to provide corrected values, categorized as small (<0.3), moderate (0.3–0.8), large (0.8–1.3), or very large (>1.3) [28].

Heterogeneity was assessed using the I^2^ statistic and chi-square (χ^2^) test and classified as low (≤40%), moderate (30–60%), substantial (50–90%), or very high (75–100%) [25]. A *p*-value < 0.10 for χ^2^ indicated statistically significant heterogeneity.

## 3. Results

### 3.1. Study Selection

**Figure 1:**
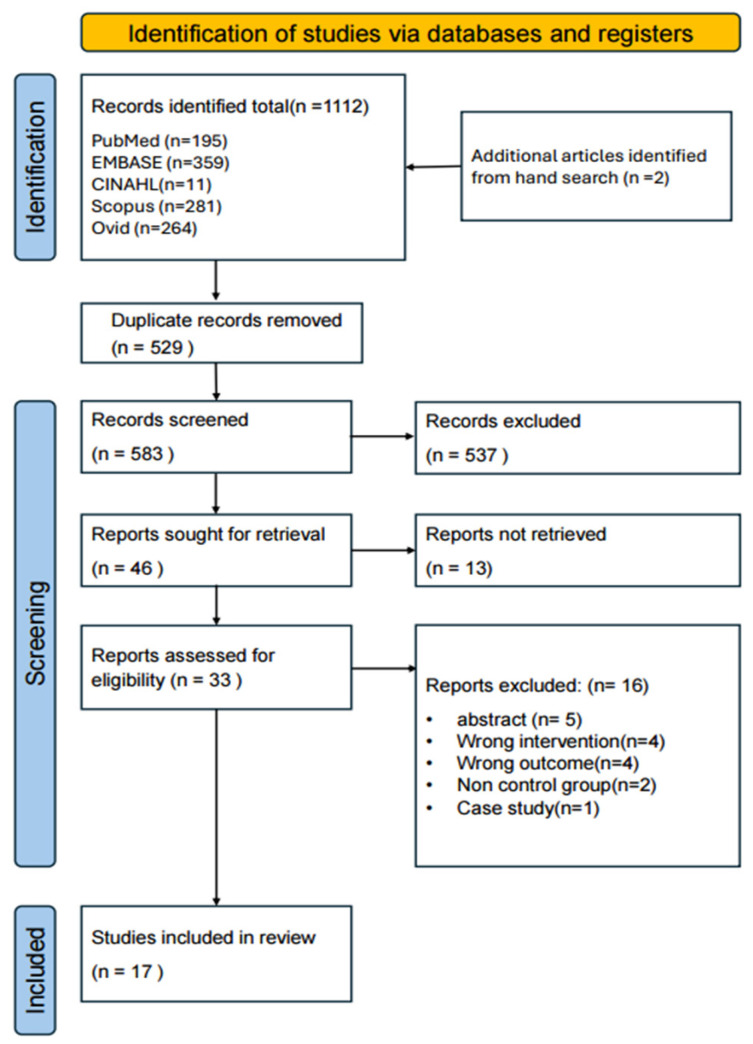
Flow diagram.

A database search initially identified 1112 articles. After excluding 529 duplicates, 583 articles remained for title and abstract screening, which excluded 537 studies and retained 46 for full-text review. Thirteen studies lacked full-text access, leaving 33 eligible studies. Of these, five were abstracts only, four used inappropriate interventions, four employed different outcome measures, two lacked control groups, and one was a case study. Overall, 16 studies were excluded after full-text review, and 17 were finally included in the review. Figure 1 shows the PRISMA flow diagram.

### 3.2. General Characteristics of the Studies

Table 1 summarizes the study characteristics. Overall, 888 participants aged 47–68 years (mean ± standard deviation: 56.36 ± 9.68) were included, comprising 554 men and 334 women. Regarding the stroke phase, one study involved patients in the acute phase, eight in the subacute phase, and eight in the chronic phase.

### 3.3. Outcomes Characteristics

Eight studies assessed gait speed, three of which measured it under two conditions: comfortable and maximal walking pace. Nine studies evaluated walking endurance using the 6 min walk test.

Balance was assessed using the BBS in nine studies and the timed up and go (TUG) test in ten studies.

### 3.4. Quality Assessment and Risk of Bias

The quality assessment results based on the PEDro scale are summarized as follows: 17 studies were included, with an average PEDro score of 6.17. Five studies report a dropout rate of 15% or higher, and 10 applied the ITT principle. Only one study was rated low quality (score ≤ 3), five were rated moderate quality (score 4–5), and 11 were rated high quality (score 6–10). All assessments were independently verified by the research team and supervised by two PhD-level physical therapists. Table 2 presents the results.

### 3.5. Meta-Analysis Results

#### 3.5.1. Gait Speed

**Figure 2:**
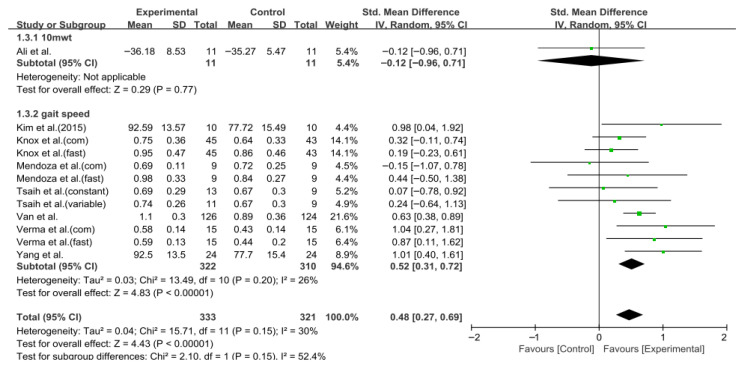
Forest plot of gait speed. Studies included: Ali et al. [30], Kim et al. (2015) [40], Knox et al. [36], Mendoza et al. [43], Tsaih et al. [35], Van et al. [44], Verma et al. [37], Yang et al. [38].

**Figure 3:**
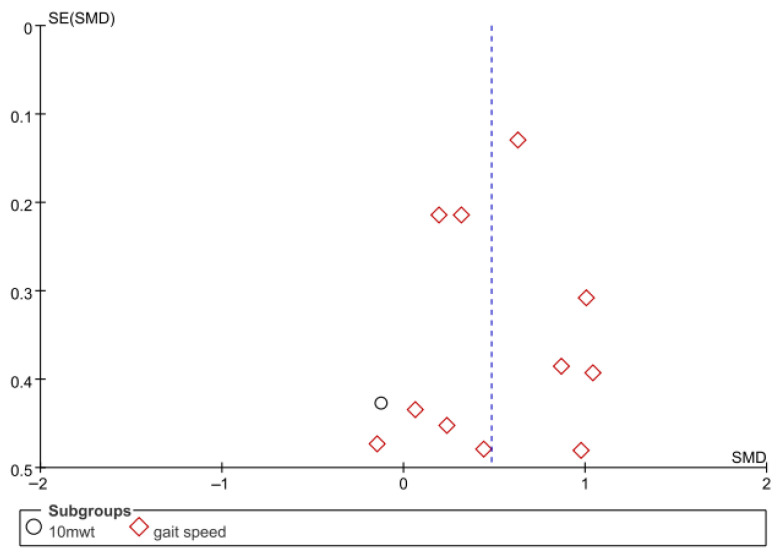
Funnel plot of gait speed.

Gait speed was assessed in eight studies comprising 654 participants. The homogeneity test revealed low heterogeneity (χ^2^ = 15.71, *p* = 0.15, I^2^ = 30%), warranting the use of a random-effects model to account for interstudy variability. The meta-analysis revealed a statistically significant improvement in the experimental group compared to the control group (Z = 4.43, *p* < 0.0001; SMD = 0.48, 95% CI: 0.27, 0.69), indicating a moderate effect size. Owing to the limited number of included studies, publication bias was not assessed, and no funnel plot analysis was performed. Figure 2 and Figure 3 show the forest and funnel plots, respectively.

#### 3.5.2. Six-Minute Walk Test

**Figure 4:**
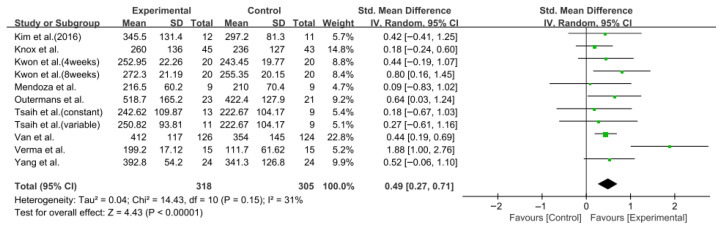
A forest plot of the 6-Minute Walk Test. Studies included: Kim et al. (2016) [31], Knox et al. [36], Kwon et al. [34], Mendoza et al. [43], Outermans et al. [45], Tsaih et al. [35], Van et al. [44], Verma et al. [37], Yang et al. [38].

**Figure 5:**
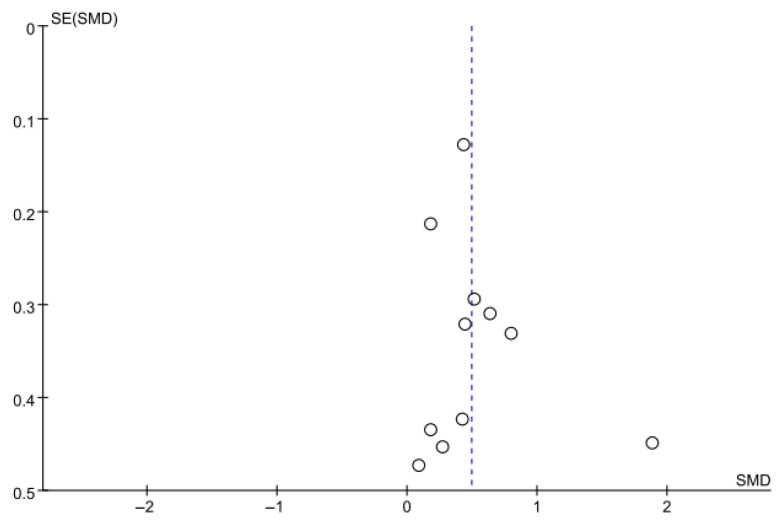
A funnel plot of the 6-Minute Walk Test.

The 6 Min Walk Test (6MWT) was assessed in nine studies comprising 623 participants. The homogeneity test revealed low heterogeneity (χ^2^ = 14.43, *p* = 0.15, I^2^ = 31%), warranting the use of a random-effects model to account for interstudy variability. The meta-analysis showed a statistically significant improvement in the experimental group compared to the control group (Z = 4.43, *p* < 0.0001; SMD: 0.49, 95% CI: 0.27, 0.71), reflecting a moderate effect size. Owing to the limited number of included studies, publication bias was not assessed, and no funnel plot analysis was performed. Forest and funnel plots are presented in Figure 4 and Figure 5, respectively.

#### 3.5.3. Berg Balance Scale

**Figure 6:**
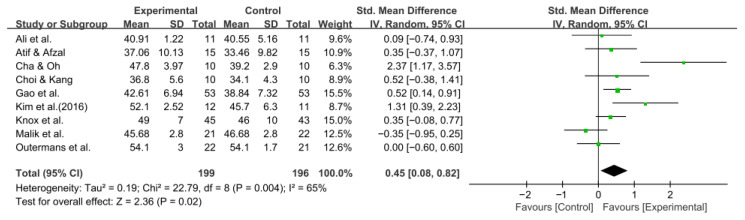
A forest plot of the Berg Balance Scale. Studies included: Ali et al. [30], Atif and Afzal [41], Cha and Oh [32], Choi and Kang [42], Gao et al. [29], Kim et al. (2016) [31], Knox et al. [36], Malik et al. [39], Outermans et al. [45].

**Figure 7:**
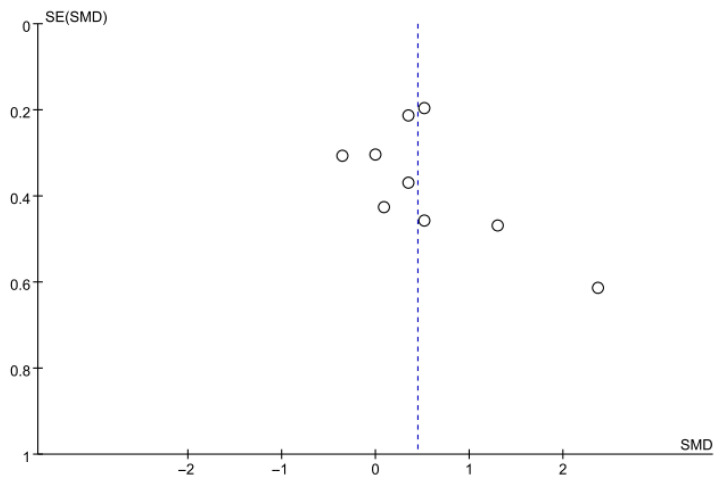
A funnel plot of the Berg Balance Scale.

Balance was assessed using the BBS in nine studies comprising 395 participants. The homogeneity test indicated high heterogeneity (χ^2^ = 22.79, *p* = 0.004, I^2^ = 65%), warranting the use of a random-effects model to account for interstudy variability. Because heterogeneity remained moderate (I^2^ = 65%), subgroup analyses were performed, and detailed results are presented in Table 3. The meta-analysis showed a statistically significant improvement in the experimental group compared with the control group (Z = 2.36, *p* = 0.02; SMD: 0.45, 95% CI: 0.08, 0.82), reflecting a moderate effect size. Owing to the limited number of included studies, publication bias was not assessed, and no funnel plot analysis was performed. Figure 6 and Figure 7 show the forest and funnel plots.

#### 3.5.4. Timed up and Go Test

**Figure 8:**
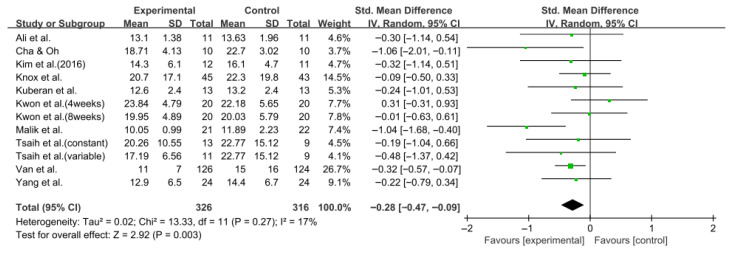
A forest plot of the Timed Up and Go test. Studies included: Ali et al. [30], Cha and Oh [32], Kim et al. (2016) [31], Knox et al. [36], Kuberan et al. [33], Kwon et al. [34], Malik et al. [39], Tsaih et al. [35], Van et al. [44], Yang et al. [38].

**Figure 9:**
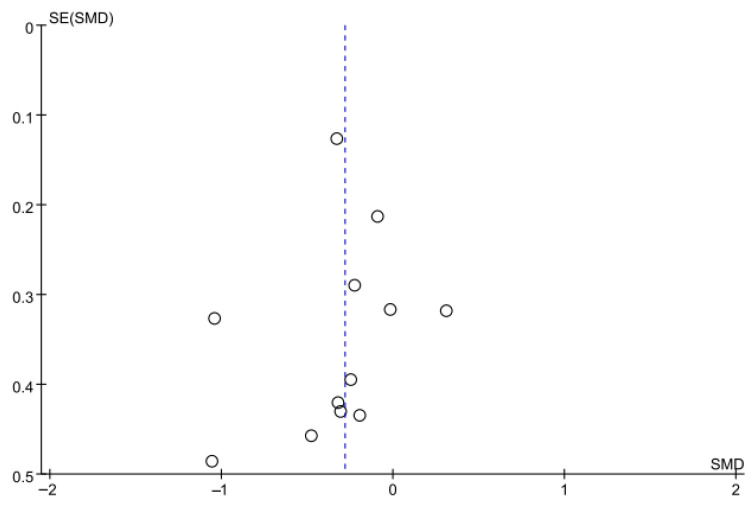
A funnel plot of the Timed Up and Go test.

Balance was also evaluated using the TUG test in 10 studies comprising 642 participants. The homogeneity test indicated low heterogeneity (χ^2^ = 13.33, *p* = 0.27, I^2^ = 17%), warranting the use of a random-effects model to account for interstudy variability. The meta-analysis revealed a statistically significant reduction in the experimental group compared with the control group (Z = 2.92, *p* = 0.003; SMD: −0.28, 95% CI: −0.47, −0.09), indicating a small effect size. Egger’s regression test (t = −0.81, *p* = 0.44) and the Trim and Fill method identified no additional studies, suggesting no publication bias. Figure 8 and Figure 9 show the forest and funnel plots.

## 4. Discussion

This systematic review and meta-analysis examined the effects of TOT on gait and balance in patients with stroke, incorporating 17 RCTs. TOT significantly improved gait speed and 6MWT performance, as well as balance outcomes measured via the BBS and the TUG test.

For gait speed, a random-effects model analysis of eight studies indicated a moderate effect. Similarly, a meta-analysis of nine studies on the 6MWT showed a moderate improvement. These findings align with those of previous research demonstrating that TOT and circuit-based gait training significantly improve gait speed and endurance. Jeon et al. (2015) report that TOT improves overall functional recovery and gait-related outcomes [46], while Schröder et al. (2019) indicate that early repetitive gait training improves gait performance during initial rehabilitation [47]. Similarly, the results of this study regarding gait speed and the 6MWT suggest that intervention timing and intensity may affect the magnitude of improvement. Furthermore, Bonini-Rocha et al. (2018) report that circuit-based gait training was more effective than conventional gait rehabilitation in improving gait speed and endurance [48]. A network meta-analysis by Lyu et al. (2023) also reports that TOT significantly improves gait function and outperforms conventional gait training in enhancing functional mobility [8].

These consistent findings suggest that task-oriented approaches improve gait-related fitness, mobility, and environmental adaptability. This indicates that such training may enhance walking endurance beyond mere speed improvements. These findings support the mechanism that gait rehabilitation promotes motor learning and neuromuscular adaptation through repeated, task-directed movements [49]. However, because the included patients were at different stages of stroke recovery, variations in intervention timing and intensity (e.g., number of sessions, total treatment time) may have contributed to outcome differences. Therefore, future research should systematically examine these moderating effects through subgroup analyses or meta-regression.

This study assessed balance using the BBS and TUG test. The BBS showed a moderate effect, while the TUG showed a significant reduction in completion time across 10 studies involving 642 participants. Egger’s regression test and the Trim and Fill analysis revealed no evidence of publication bias. These findings align with those of previous studies. Zhou et al. (2024) report significant improvements in BBS and TUG in a meta-analysis of 29 RCTs examining the effects of exercise on balance in patients with stroke [50]. Similarly, Li et al. (2019) report that various balance interventions significantly enhance postural control and mobility during stroke recovery [51]. Conversely, Li et al. (2025) report significant improvements in BBS but no notable changes in TUG in their meta-analysis of home-based exercise interventions [52]. Compared with previous findings, the BBS and TUG effects in this study are directionally consistent but differ in magnitude and significance. These differences likely result from variations in participant characteristics, heterogeneity in intervention components, and differences in assessment timing across studies. In particular, balance and mobility improvements may be more limited in chronic-phase patients than in acute-phase patients owing to reduced neuroplasticity, which could explain the variability in TUG outcomes.

However, the BBS showed high heterogeneity. Subgroup analysis revealed significant differences based on stroke phase. This variation likely stemmed from differences in patient status (acute vs. chronic phase) and the timing of initial intervention across studies. Specifically, while some studies emphasized static balance tasks, others included dynamic tasks such as weight shifting and maintaining balance during walking, indicating notable differences in training intensity and stimuli. These factors likely contribute to the variability in effect size and overall heterogeneity. Furthermore, since the effectiveness of balance training varies with intervention frequency and duration, these variables may also have influenced the BBS outcomes.

In this study, interventions lasting 60 min or less and more than three sessions per week were more effective in improving BBS scores. This suggests that higher intervention intensity and frequency may play a key role in enhancing balance recovery. Similarly, Li et al. (2025) report the greatest balance improvements in programs lasting at least 8 weeks with three or more sessions weekly [52]. Conversely, some studies may have underestimated effects due to short intervention durations or evaluations conducted only immediately after intervention. Nonetheless, these findings should be interpreted cautiously, given the limited number of studies included in this meta-analysis.

Balance recovery after stroke depends on the integration of visual, somatosensory, and vestibular inputs, and because stroke often disrupts vestibular processing or sensory reweighting, task-oriented activities involving head movements, gait transitions, and dynamic postural adjustments may help stimulate vestibular pathways [53]. This enhanced multisensory integration may partly explain the improvements observed in BBS and TUG outcomes [54].

In summary, this study demonstrates that the interventions improve balance and mobility, as reflected in the BBS and TUG outcomes. Clinically, TOT that incorporates both static and dynamic balance components may be more effective, and ensuring adequate intervention duration and intensity is crucial. Additionally, tailoring the timing of balance training to the recovery stage of the patient may be a key determinant of its effectiveness.

These findings suggest that interventions targeting gait and balance substantially enhance motor function recovery in patients with stroke. First, incorporating balance-challenging tasks into gait training or combining both approaches may maximize therapeutic benefits. Second, response patterns may vary across recovery phases (acute, subacute, chronic), highlighting the importance of appropriate intervention timing and participant selection. Third, factors such as training intensity, session frequency, and program duration may influence outcomes, underscoring the need for standardized and evidence-based intervention protocols.

Participants in the included studies were randomly assigned to experimental or control groups, with a mean PEDro score of 6.17, indicating high methodological quality (PEDro scale: 6–10). The PEDro score assesses the internal validity and methodological rigor of clinical trials, and the average score in this review exceeded the mean in the PEDro database of 5 [55]. These findings suggest that the internal validity of this review is well established. However, this study has several limitations. First, the limited number of RCTs reduces the robustness of the findings, warranting cautious interpretation. Second, the high heterogeneity observed in the BBS analysis may reflect insufficient control of confounding variables or the influence of unreported factors. Third, most studies report only immediate post-intervention outcomes, providing limited evidence of long-term effects after treatment cessation. Fourth, the small number of available studies limited meta-regression analysis, restricting subgroup analyses to exploratory interpretation. Fifth, the coexistence of gait-focused, balance-focused, and combined interventions complicates direct comparisons of their effects. Therefore, future research should incorporate more high-quality RCTs to examine the influence of intervention components (intensity, frequency, modality), integrated gait and balance strategies, long-term retention effects, and differences in response across patient characteristics.

## 5. Conclusions

This systematic review and meta-analysis, synthesizing 17 RCTs involving patients with stroke, demonstrated that TOT significantly improves gait speed, walking endurance, and balance. Moderate effect sizes were observed for gait outcomes, including gait speed and the 6MWT, while balance measures such as the BBS and the TUG test also showed significant improvements. These findings suggest that a TOT is more effective than simple repetitive exercise in promoting functional recovery and enhancing environmental adaptability. Furthermore, intervention intensity (number of sessions and weekly frequency) and stroke phase (acute vs. chronic) were identified as key factors influencing balance outcomes. Therefore, in clinical practice, task-oriented programs should integrate gait and balance components tailored to the recovery phase and target functions of the patients. Future research should focus on long-term follow-up outcomes, the effects of specific intervention components, and the development of standardized training protocols. These efforts will help enhance functional independence and quality of life in patients with stroke.

## Figures and Tables

**Table 1:** The characteristics of the studies included in the meta-analysis.

Study (Year)	Age (Years, Mean ± SD)	Sample Size (Sex (M/F))	Phase of Stroke	Intervention	Outcomes
Gao et al. (2024) [29]	E: 67.9 ± 7.0 C: 65.5 ± 6.4	E: 53 (26/27) C: 53 (31/22)	Subacute	E: essential rehabilitation treatment and Task-Oriented Biomechanical Perception-Balance training C: essential rehabilitation training treatment	BBS, FMA
Ali et al. (2020) [30]	Total: 60.81	E: 11 (7/4) C: 11 (6/5)	Subacute	E: group task-specific training C: individual task-specific training	BBS, TUG, 10MWT
Kim et al. (2016) [31]	E: 50 ± 9.3 C: 54 ± 7.1	E: 15 (8/7) C: 15 (10/5)	Chronic	E: group task-oriented circuit training C: individual task-oriented circuit training	BBS, TUG, 6MWT
Cha and Oh (2016) [32]	E: 60 ± 3.19 C: 58.6 ± 4.08	E: 10 (4/6) C: 10 (5/5)	Chronic	E: task-oriented exercise program with a mirror C: task-oriented exercise program	BBS, TUG
Kuberan et al. (2017) [33]	E: 58.82 ± 9.12 C: 60.07 ± 7.56	E: 13 (9/4) C: 13 (9/4)	Chronic	E: task-oriented training with sensory input manipulations and provision of sensory conflict for the trunk and lower limb C: conventional physiotherapy program	TUG
Kwon et al. (2015) [34]	E: 50.7 ± 15.16 C: 47.15 ± 18.65	E: 20 (14/6) C: 20 (12/8)	Chronic	E: task-oriented treadmill walking training C: conventional treadmill walking training	TUG, 6MWT
Tsaih et al. (2018) [35]	E1: 48.6 ± 12.6 E2: 55.5 ± 12.4 C: 56.1 ± 9.0	E1: 11 (10/1) E2: 13 (9/4) C: 9 (7/2)	Chronic	E1: variable-practice EMGBFB and general PT E2: constant-practice EMGBFB and general PT C: upper extremity exercise and general PT	Gait speed, TUG, 6MWT
Knox et al. (2018) [36]	E: 51 ± 15 C: 48 ± 14	E: 51 (25/26) C: 48 (22/26)	Subacute	E: task intervention C: stroke management	Gait speed (CV, FV), BBS, TUG, 6MWT
Verma et al. (2011) [37]	E: 53.27 ± 8.53 C: 55.07 ± 6.8	E: 15 (10/5) C: 15 (12/3)	Acute	E: motor imagery and task-oriented circuit class training C: lower extremity rehabilitation program	Gait speed (CV, FV), 6MWT
Yang et al. (2006) [38]	E: 56.8 ± 10.2 C: 60.0 ± 10.4	E: 24 (16/8) C: 24 (16/8)	Chronic	E: task-oriented progressive resistance strength training C: did not receive any rehabilitation training	Gait speed, 6MWT, TUG
Malik et al. (2021) [39]	Total: 40–70	E: 26 C: 26 Total: 52 (36/16)	Subacute	E: task-oriented training and exercise gaming C: task-oriented training	TUG, BBS
Kim et al. (2015) [40]	E: 58.53 ± 11.83 C: 61.24 ± 8.73	E: 10 (4/6) C: 10 (6/4)	Subacute	E: routine therapy and tilted table with task-oriented training C: routine therapy and tilted table	Gait speed
Atif and Afzal (2023) [41]	-	E: 15 C: 15 Total: 30 (18/12)	Subacute	E: task-oriented walking C: conventional therapy	BBS
Choi and Kang (2015) [42]	E: 61.5 ± 7.2 C: 66.4 ± 9.3	E: 10 (4/6) C: 10 (4/6)	Chronic	E: task-oriented training C: general physical therapy	BBS
Mendoza et al. (2021) [43]	E: 47.2 ± 8.8 C: 49 ± 11.2	E: 9 (9/0) C: 9 (7/2)	Chronic	E: task-oriented circuit class training C: circuit class training focused on specific impairments	Gait speed (CV, FV), 6MWT
Van et al. (2012) [44]	E: 56 ± 10 C: 58 ± 10	E: 126 (82/44) C: 124 (80/44)	Subacute	E: task-oriented circuit training C: outpatient physiotherapy	Gait speed (CV), 6MWT, TUG
Outermans et al. (2010) [45]	E: 56.8 ± 8.6 C: 56.3 ± 8.6	E: 23 (19/4) C: 21 (17/4)	Subacute	E: high-intensity task-oriented training C: low-intensity physiotherapy	6MWT, 10MWT, BBS

E, Experimental group; C, Control group; BBS, Berg Balance Scale; 10MWT, 10 m walking test; 6MWT, 6 min walking test; TUG, Timed up and go test; CV, Comfortable velocity; FV, Fast velocity; PT, Physiotherapy; FMA, Fugl-meyer assessment; EMGBFB, Electromyographic biofeedback.

**Table 2:** An assessment of methodological quality using the PEDro scale.

Study (Year)	1	2	3	4	5	6	7	8	9	10	11	Score
Gao et al. (2024) [29]	Y	Y	N	Y	N	N	N	Y (0%)	Y	Y	Y	6
Ali et al. (2020) [30]	Y	Y	N	N	N	N	N	Y (0%)	Y	Y	Y	5
Kim et al. (2016) [31]	Y	Y	N	N	N	N	N	N (23%)	N	Y	Y	3
Cha and Oh (2016) [32]	Y	Y	N	Y	N	N	Y	N (20%)	N	Y	Y	5
Kuberan et al. (2017) [33]	Y	Y	N	Y	N	N	Y	Y (0%)	Y	Y	Y	7
kwon et al. (2015) [34]	Y	Y	N	Y	N	N	N	Y (9%)	N	Y	Y	5
Tsaih et al. (2018) [35]	Y	Y	N	Y	N	N	Y	Y (0%)	Y	Y	Y	7
Knox et al. (2018) [36]	Y	Y	Y	Y	N	Y	Y	Y (11%)	N	Y	Y	8
Verma et al. (2011) [37]	Y	Y	Y	Y	Y	N	Y	Y (3%)	Y	Y	Y	9
Yang et al. (2006) [38]	Y	Y	Y	Y	N	N	Y	Y (0%)	Y	Y	Y	8
Malik et al. (2021) [39]	Y	Y	Y	Y	N	N	Y	N (17%)	N	Y	Y	6
Kim et al. (2015) [40]	Y	Y	N	Y	N	N	Y	N (18%)	N	Y	Y	5
Atif and Afzal (2023) [41]	Y	Y	N	N	N	N	N	Y (0%)	Y	Y	Y	5
Choi and Kang (2015) [42]	Y	Y	N	Y	N	N	N	Y (0%)	Y	Y	Y	6
Mendoza et al. (2021) [43]	Y	Y	Y	Y	N	N	Y	Y (0%)	Y	Y	Y	8
Van et al. (2012) [44]	Y	Y	Y	N	N	N	Y	Y (3%)	N	Y	Y	6
Outermans et al. (2010) [45]	Y	Y	Y	Y	N	N	N	N (27%)	Y	Y	Y	6

Y, Yes; N, No.

**Table 3:** A subgroup analyses of task-oriented training on the Berg Balance Scale in nine trials.

Category	Subgroup	No. of Trials	Sample Size	SMD (d) [95% CI]	Heterogeneity *p*-Value of Chi-Square Test (I^2^)	Overall Effect Z Value (*p*-Value)	Subgroup Diff. *p*-Value
Phase of stroke	Acute	0	0				
	Subacute	6	332	0.22 [−0.04, 0.48]	6.82 (27%)	1.63 (0.10)	0.03 *
	Chronic	3	63	1.33 [0.34. 2.32]	5.92 (66%)	2.64 (0.008 *)	
During session	≤60 min	5	198	0.64 [0.10, 1.18]	10.29 (61%)	2.33 (0.02 *)	0.32
	>60 min	4	197	0.25 [−0.29, 0.80]	9.70 (69%)	0.91 (0.36)	
Weekly frequency	≤3 times/week	6	249	0.23 [−0.14, 0.61]	9.92 (50%)	1.21 (0.22)	0.14
	>3 times/week	3	146	1.00 [0.06, 1.95]	8.36 (76%)	2.08 (0.04 *)	
Duration of trial	≤6 weeks	5	128	0.77 [0.00, 1.53]	15.86 (75%)	1.96 (0.05)	0.24
	>6 weeks	4	267	0.26 [−0.10, 0.61]	5.83 (49%)	1.43 (0.15)	

* *p* < 0.05, SMD, standardized mean difference; CI, confidence interval.

## Data Availability

The datasets generated during and/or analyzed during the current research are available from the corresponding author upon reasonable request.

## Supplementary Materials

The following supporting information can be downloaded at: https://www.mdpi.com/article/10.3390/jcm14248766/s1, Table S1: PRISMA 2020 Checklist.
